# PFTK1 Promotes Gastric Cancer Progression by Regulating Proliferation, Migration and Invasion

**DOI:** 10.1371/journal.pone.0140451

**Published:** 2015-10-21

**Authors:** Lei Yang, Jia Zhu, Hua Huang, Qichang Yang, Jing Cai, Qiuhong Wang, Junya Zhu, Mengting Shao, Jinzhang Xiao, Jie Cao, Xiaodan Gu, Shusen Zhang, Yingying Wang

**Affiliations:** 1 Department of Oncology, Nantong Tumor Hospital, Nantong, Jiangsu, China; 2 Department of Pathogen Biology, Jiangsu Province Key Laboratory for Inflammation and Molecular Drug Target, Medical College of Nantong University, Nantong 226001, Jiangsu, China; 3 Department of Pathology, Affiliated Hospital of Nantong University, Nantong, Jiangsu, China; 4 Department of Pathology, Nantong first people's hospital, Nantong, Jiangsu, China; The University of Texas MD Anderson Cancer Center, UNITED STATES

## Abstract

PFTK1, also known as PFTAIRE1, CDK14, is a novel member of Cdc2-related serine/threonine protein kinases. Recent studies show that PFTK1 is highly expressed in several malignant tumors such as hepatocellular carcinoma, esophageal cancer, breast cancer, and involved in regulation of cell cycle, tumors proliferation, migration, and invasion that further influence the prognosis of tumors. However, the expression and physiological significance of PFTK1 in gastric cancer remain unclear. In this study, we analyzed the expression and clinical significance of PFTK1 by Western blot in 8 paired fresh gastric cancer tissues, nontumorous gastric mucosal tissues and immunohistochemistry on 161 paraffinembedded slices. High PFTK1 expression was correlated with the tumor grade, lymph node invasion as well as Ki-67. Through Cell Counting Kit (CCK)-8 assay, flow cytometry, colony formation, wound healing and transwell assays, the vitro studies demonstrated that PFTK1 overexpression promoted proliferation, migration and invasion of gastric cancer cells, while PFTK1 knockdown led to the opposite results. Our findings for the first time supported that PFTK1 might play an important role in the regulation of gastric cancer proliferation, migration and would provide a novel promising therapeutic strategy against human gastric cancer.

## Introduction

Gastric cancer is the fourth most common malignant tumor and the second leading cause of cancer-related mortality in all kinds of cancers worldwide [1]. It is difficult to cure unless it is found at an early stage [2]. Owing to the lack of specificity, early diagnosis rate is low and the majority of gastric cancer patients are at middle-late stage when diagnosis. 40%–60% patients with gastric cancer received gastric cancer radical operation will often have postoperative recurrence and metastasis, these characteristics seriously affect the long-term survival in patients with gastric cancer [3,4]. Despite great advancement of new diagnosis and treatment strategies of gastric cancer, the exact molecular mechanisms of gastric cancer remains poorly understand. Thus, the identification of the molecular mechanism during gastric cancer progression and metastasis may provide patients with novel diagnostic and therapeutic strategies.

PFTK1 (also known as PFTAIRE1, CDK14) is a novel member of Cdc2-related serine/threonine protein kinases that is first identified in the mouse nervous system and is a crucial regulator of cyclins and cell cycle [5,6]. Few studies have been performed to characterize its physiological function or biological importance. It is reported that PFTK1 is highly expressed in brain, pancreas, kidney, and ovary. CDKs bind to specific cyclin box to form functional protein kinase complexes and regulated in part by its subcellular localization [7]. For instance, Cyclin Y, a novel membrane-associated cyclin, interacts with PFTK1 enhances PFTK1 kinase activity and recruits PFTK1 to the plasma membrane [8]. PFTK1 interacts with Cyclin B2 co-localization in the nucleus in hepatocellular carcinoma [9]. The fundamental function of PFTK1 is reported as a cyclin-dependent kinase (CDK) regulating cell cycle progression and cell proliferation by specifically interacting with members of cyclin proteins such as Cyclin D3 (CCND3), Cyclin Y (CCNY) and forms a ternary complex with the cell cycle inhibitor p21, thus phosphorylates the tumor suppressor Rb for G1/S transition [10]. Knockout Cyclin Y in glioma cell lines makes the cell cycle blocked in S period [11]. Cyclin Y interacts with PFTK1 adjust M phase of mitosis [12]. These findings together implicate that PFTK1 may function as a tumor promoter via regulating cell cycle. Meanwhile, several studies demonstrate that PFTK1 also has other important functions. PFTK1 modulates oligodendrocyte differentiation via PI3K/AKT pathway [13]. Recently PFTK1 confers HCC cell motility through inactivating the actin-binding motile suppressing function of TAGLN2 via phosphorylation [14]. PFTK1-mediated phosphorylation enables association of CaD to F-actin filaments, resulting in enhancing polymerization of the actin stress fibers, thus promoting cell migration and invasion in HCC cells [15,16]. Overexpression of PFTK1 may confer a motile phenotype in malignant hepatocytes [9]. In agreement with the molecular findings, CCNY and/or PFTK1 alone can activate noncanonical Wnt signaling to enhance cell motility in HCC cells [17]. All of these studies imply that PFTK1 may be involved in the cell proliferation and motility, however the expression and significance of PFTK1 in gastric cancer cells are still obscure.

In our study, we aimed to conduct a comprehensive analysis of PFTK1 expression and its prognosis role in gastric cancer cells. We examined the expression of PFTK1 and its association with clinical characteristics and Ki-67 by Western blot and immunohistochemistry (IHC). Our study showed that PFTK1 enhanced proliferation, migration and invasiveness of gastric cancer cells by CCK-8, flow cytometry analyses, colony formation analyses, transwell assay, affected the expression of related proteins. These findings were firstly reported in gastric cancer cells and may provide a novel insight into developing experimental therapies in gastric cancer.

## Materials and Methods

### Tissue samples

One hundred and sixty-one gastric cancer tissue samples and matched adjacent noncancer tissues were selected from patients who underwent surgery between 2007 and 2013 at Department of Pathology, Nantong Tumor Hospital. These tissues were stored at −80°C immediately after surgical removal. For histological examination, all gastric tissue samples were fixed in 10% buffered formalin and embedded in paraffin for sectioning. Resected specimens were classified according to the Seventh Edition of the TNM Classification for Gastric Cancer [1].All the clinicopathological information included age, gender, infiltration depth, differentiation status, lymph node invasion, nerve invasion and TNM stage. The approval of removed tissues for research purposes was obtained from the Ethics Committee of Nantong Tumor Hospital. Signed informed consent was also obtained from each patient. The main clinical and pathologic variables were summarized in Table 1.

**Table 1:** PFTK1 and Ki-67 expression and clinicopathologic characteristics on 161 gastric specmens.

Characteristics	Total	PFTK1 expression		χ^2^ value	P value
		Low	High		
Age				0.223	0.636
≤ 60	68	26	42		
60	93	39	54		
Gender				1.349	0.246
Female	53	18	35		
Male	108	47	61		
Tumor grade				9.421	0.009*
Well	16	11	5		
Moderate	82	36	46		
Poor and others	63	18	45		
Infiltration depth				12.295	0.002*
Inferiormucousemb-rane layer	18	14	4		
Muscular layer	67	26	41		
Serous layer	76	25	51		
TNM stage				4.853	0.028*
I-II	58	30	28		
III-IV	103	35	68		
Lymph node				21.026	0.000*
Negative	16	15	1		
Positive	145	50	95		
Nerve invasion				0.007	0.936
Negative	96	39	57		
Positive	63	26	37		
Ki-67 expression				41.466	0.000*
Low	63	45	18		
High	98	20	78		

Statistical analyses were performed by the Pearson χ^2^ test

* P <0.05 was considered significant

### Western blot analysis

Western blot assay was used to detect some proteins [18–20]. Firstly, the gastric caner tissues and cells were cracked in lysis buffer (1 M Tris-Hcl pH 7.5, 1% Triton X-100, 10% sodium sulfate (SDS), 1% NP-40, 0.5 M EDTA, 0.5% sodium deoxycholate, 10 μg/mL aprotinin, 1 mM PMSF, 10 μg/mL leupeptin) and then centrifuged at 10,000× g for 30 min to collect the supernatant. The supernatant was diluted in 2×SDS loading buffer and boiled. An equivalent amount of proteins from each sample were electrophoresed by 10% sodium dodecyl sulfate-polyacrylamide gel electrophoresis (SDS-PAGE) and then transferred on to a Polyvinylidene fluoride (PVDF) membrane (Millipore, Bedford, MA). After that, the membranes were blocked for about 2 h at room temperature and then incubated overnight at 4°C with the primary antibodies. After washing with Phosphate Buffered Saline (PBS) containing 0.1% Tween 20 three times, each for 5 min, the membranes were then incubated with horseradish peroxidase-linked IgG as the secondary antibody for another 2 h at room temperature. The membranes were then developed using the ECL detection systems (Imaging Technology, Ontario, Canada). The antibodies used in this study included: anti-PFTK1 (1:500, Santa Cruz Biotechnology), anti-Cyclin E (1:500, Santa Cruz Biotechnology), anti-PCNA (1:1000, Santa Cruz Biotechnology), anti-GAPDH (1:1000, Sigma), anti-β-catenin (1:1000, Santa Cruz Biotechnology), anti-Dvl1 (1:600, Santa Cruz Biotechnology), anti-Dvl2 (1:600, Santa Cruz Biotechnology), anti-Naked1 (1:500, Cell Signaling), anti-MMP2 (1:500, Santa Cruz Biotechnology).

### Immunohistochemical analyses

In brief, tissue sections were dewaxed in xylene and rehydrated through graded ethanols. Then the sections quenched in 3% methanolic peroxide for about 10 min to block endogenous peroxidase activity. By high pressure and temperature in an autoclave, its immunoreactivity was enhanced in 0.1 M citrate buffer for 3 min. Tissue sections were incubated with the anti-PFTK1 (1:100, Santa Cruz Biotechnology) and anti-Ki-67 (1:400, Santa Cruz Biotechnology) for 120 min at room temperature. According to the manufacturer’s instructions, after washing in phosphate-buffered saline (PBS), tissues were incubated with horseradish peroxidase-conjugated anti-mouse Ig polymer as a second antibody (Envision kit, Dako) for 20 min at room temperature. Finally, slides were counterstained with hematoxylin, dehydrated, and mounted in resin mount. To quantify PFTK1 and Ki-67 protein expression, both the extent of immunoreactivity and the intensity were evaluated and scored. Five high-power fields were chosen randomly for each section and at least 300 cells were counted per field. For statistical analysis of PFTK1, each slide was evaluated using a semiquantitative scoring system for both the intensity of the stain and the percentage of positive malignant cells. The cells were scored as follows:1 (≤25% tumor cells stained), 2 (26%-50% tumor cells stained), 3 (51%-75% tumor cells stained), 4 (76%-100% tumor cells stained). For density evaluation:1 weak staining; 2 moderate staining; 3 strong staining. Then we multiplied the two scores and classified them into two groups: low expression and high expression. As for statistical analysis of Ki-67, <50% tumor cells stained as low expression and ≥50% tumor cells stained as high expression. In order to avoid technical errors, staining was repeated three times and similar results were obtained.

### Cell cultures and transient transfection

MGC803 and HGC27 were gastric cell lines,which were purchased from the cell library of the Chinese Academy of Sciences. Both were cultured in RPMI-1640 medium (GIBCO-BRL, Grand Island, NY, USA). SGC7901 and GES1 were another gastric cell lines, which were kindly provided by the Department of Pathology Research of Nantong University. SGC7901 and GES1 were cultured in DMEM medium (GIBCO-BRL, Grand Island, NY, USA). All the medium were made with 10% fetal bovine serum (GIBCO-BRL, Grand Island, NY, USA) supplemented with 2 mM L-glutamine,100 U/ml penicillin-streptomycin mixture (GIBCO-BRL, Grand Island, NY, USA), at 37°C and 5% CO_2_. PFTK1 small-interfering RNA (siRNA) were designed and synthesized by Shanghai Genechem (China). The siRNA targeting PFTK1 sequences were as follows: 5’-GTTCATTCTTTACCACATT-3’, 5’-AGGTTGCATCTTTGTTGAA-3’,5’-AAAGAGTCACCTAAAGTTA-3’,5’-ACCCATACAGGAAATCCAA-3’. The Flag-PFTK1 plasmid was purchased from Shanghai Genechem (China). Lipofectamine 2000 transfection reagent (Life Technologies) was used for cells transfection following the manufacturer’s instructions.

### Flow cytometric analysis

Firstly, all gastric cell lines were fixed in 70% ethanol overnight at −20°C. second, after washed by citrate-phosphate buffer and PBS, the cells were incubated with 1 mg/ml RNaseA for 30 min at 37°C. Then, the cells were stained with 50 μg/ml propidium iodide in Phosphate Buffered Saline (PBS)-Triton for another 20 min at 4°C. Finally, the cells were analyzed by using a Becton Dickinson flow cytometer BD FACScan (San Jose,CA) and CellQuest acquisition analysis programs. The experiment was performed three times.

### Colony formation assays

MGC803 cells were cultivated in six-well culture plates (Corning inc, Corning NY) at a density of 200 cells/well after transfecting PFTK1#siRNA and Flag-PFTK1 according to the manufacturer’s instructions. After two weeks, the cell colonies (≥50 cells/colony) were counted by staining with 0.5% crystal violet.

### Cell proliferation assays

MGC803 cells which included normal, transfection of PFTK1#siRNA and Flag-PFTK1 were seeded onto 96-well cell culture cluster plates (Corning inc, Corning NY) at a density of 2×10^4^ cells/well in 100 μl culture and grown overnight. According to the manufacturer’s instructions, cells were measured using a commercial CCK-8 (Dojindo, Kumamoto, Japan). CCK-8 reagents were added to each well for 2 h incubation at 37°C. The absorbance was read at the wavelength of 450 nm in an automated plate reader. The experiments must repeat at least three times.

### Wound-healing assay

MGC803 cells were cultivated in six-well culture plates (Corning inc, Corning NY) at a density of 5×10^5^ cells/well and grown to confluence overnight. After transfected 48 hours, cells were incubated with serum free medium. A line was scratched within the conflent cell layer using the fie end of a 10 ml pipette tip (time 0) and cells were washed with PBS. Images of migrating cells were sequentially taken after 24 h, 48 h during closure of the wounded region.

### Trans-well migration assay

Cell migration assay was performed using transwell chamber (8.0 lm pore size; Costar, Cambridge, NY, USA). About 1×10^5^ cells/ml were seeded in the upper chambers in medium, and RPMI-1640 was added to the bottom chambers. After 24 h, the top cells which were not migrated were removed and the bottom cells which were migrated were fixed with paraformaldehyde and stained with crystal violet. The number of migrating cells in 5 fields was counted under 200× magnification, and the means for each chamber were determined. All experiments were repeated three times.

### Trans-well invasion assay

Cell migration assay was performed using transwell chamber(8.0 lm pore size; Costar, Cambridge, NY, USA). BD MatrigelTM Basement Membrane Matrix was added to the upper chamber for 1 h at 37°C. Then, 500 μl medium containing chemotactic factor was placed in the lower chamber. About 1×10^5^ cells/ml were seeded in the upper chambers at 37°C for 24 h.Cells were then fixed with paraformaldehyde and stained with crystal violet. All experiments were repeated three times.

### Statistical analysis

All statistical analysis was were carried out using the SPSS statistics 19 software package. The PFTK1, Ki-67 expression and the clinicopathologic features were analyzed using the χ^2^ test. Overall survival curves were calculated with the Kaplan–Meier method and were tested with the log-rank test. Multivariate analysis was analyzed by Cox's proportional hazards model, the risk ratio and its 95% confidence interval were recorded for every marker. The values were expressed as mean±SE, and *P*< 0.05 was considered statistically significant.

## Results

### PFTK1 is overexpressed in gastric tumor tissues

**Fig 1:**
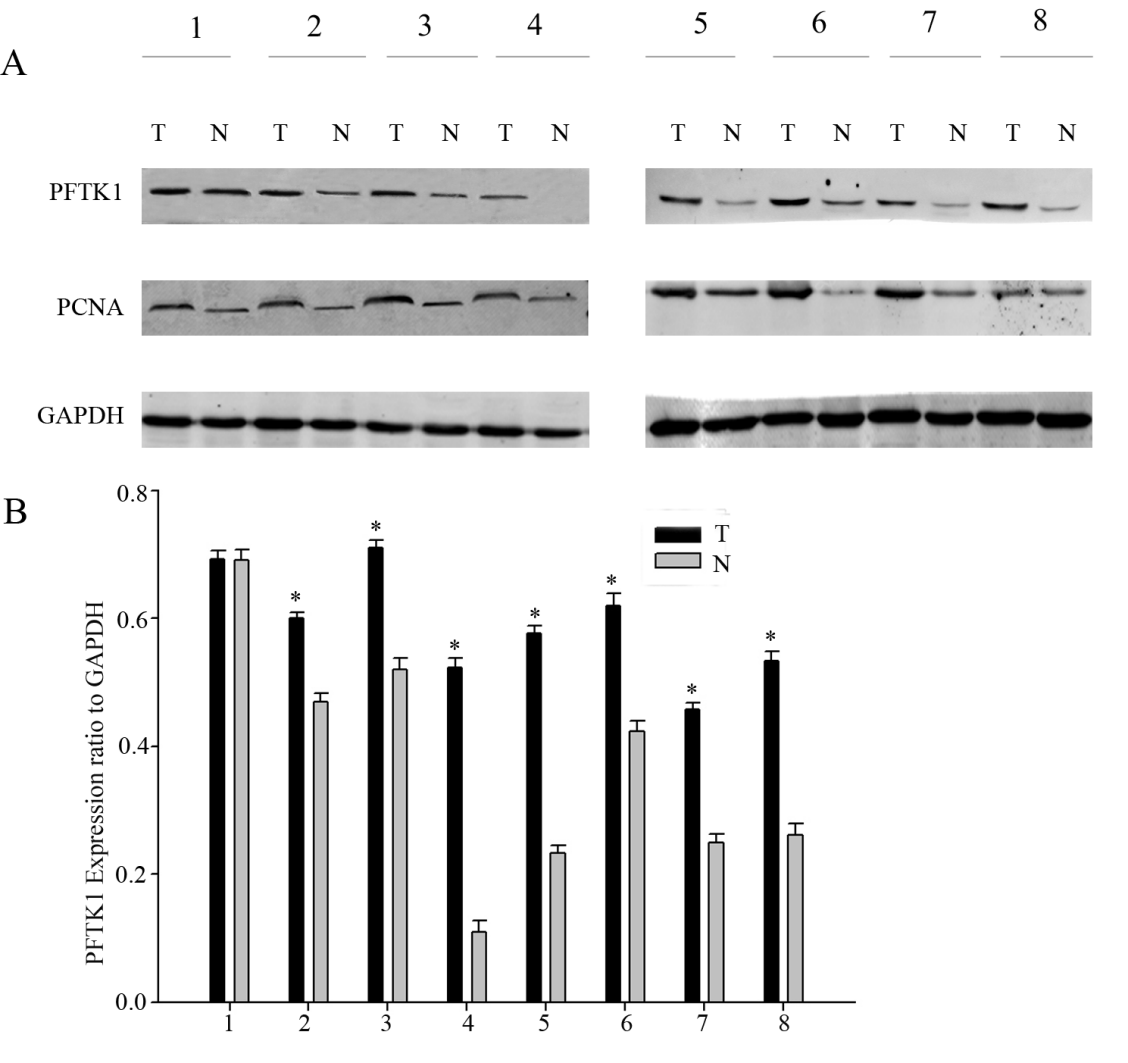
PFTK1 and PCNA expression in human gastric cancer by Western blot analysis. (A) The protein level of PFTK1 was high in eight representative paired samples of gastric cancer tissue (T) compared with nontumorous adjacent tissues (N). GAPDH was used as a loading control. (B) The bar chart showed the ratio of PFTK1 protein to GADPH. Mean±SD of three independent experiments. (*P<0.05 compared with control nontumorous adjacent tissues).

**Fig 2:**
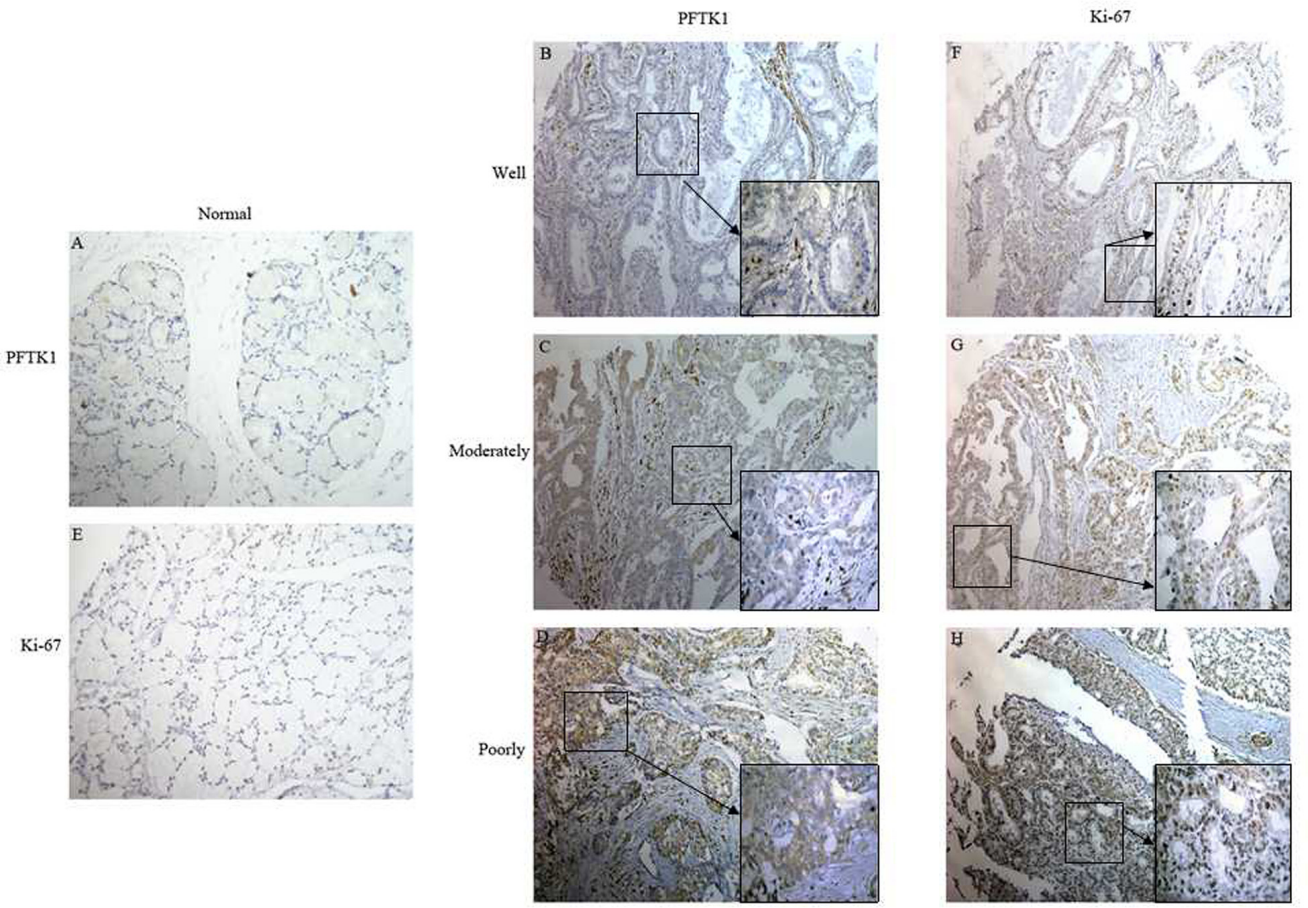
Representative photos of PFTK1, Ki-67 expressions in 161 gastric cancer tissues by IHC. (A -H) Paraffinembedded tissue sections were stained with antibodies for PFTK1 and Ki-67 and counterstained with hematoxylin. (A,E) negative staining of PFTK1, Ki-67 in adjacent normal tissues (×200); (B,F) weak staining of PFTK1, Ki-67 in well differentiated gastric cancer tissues; (C,G) moderate staining of PFTK1, Ki-67 in moderate differentiated gastric cancer tissues; (D,H) strong staining of PFTK1, Ki-67 in poor differentiated gastric cancer tissues, amplification (×100, ×400).

**Fig 3:**
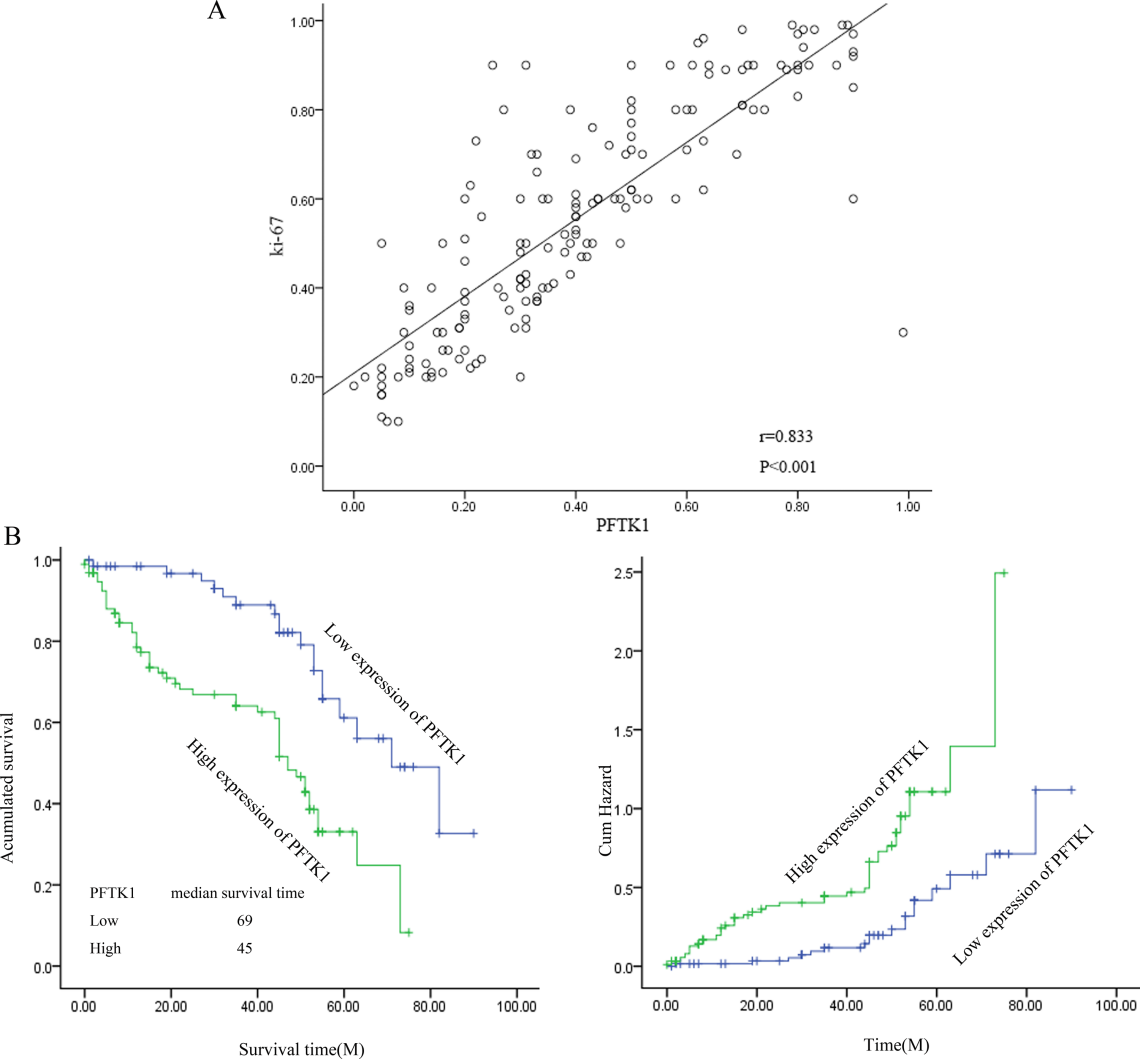
The relation between PFTK1 and clinical characteristics. (A) Correlation between PFTK1 and Ki-67 expression in gastric cancer tissues.The correlation between PFTK1 and Ki-67 expression was evaluated by Pearson correlation test (r = 0.833, *P*<0.001). (B) Kaplan–Meier survival analysis of 161 gastric cancer patients based on PFTK1 expression level. According to the PFTK1 percentages, patients were divided into high PFTK1 expressers and low PFTK1 expressers. And the median survival time and hazard ratio was showed. Patients in the high-expression PFTK1 group had a significantly shorter overall survival (*P*< 0.01).

It is reported that PFTK1 is an important cyclin dependent kinase which can regulate cell cycle, but the research on gastric cancer has never been studied. Since overexpression of PFTK1 was found in liver cancer and esophageal cancer compared to normal tissues. So it is interesting to analysis the expression of PFTK1 in gastric tumor tissues and its coorelation with proliferating cell nuclear antigen (PCNA). Firstly, we examined the expression of PFTK1 in 8 pairs of gastric tumor tissues and their corresponding nontumorous gastric mucosal tissues by Western blot. As shown in Fig 1, compared with adjacent normal tissues, upregulation of PFTK1 was detected in all eight gastric tissues in accord with PCNA. In order to confirm the clinical significance of PFTK1 in gastric cancer progression, immunohistochemical (IHC) analyses was used to observe the expression of PFTK1 protein in 161 gastric cancer tissues. As expected, the expression of PFTK1 and tumor grade were negatively related. PFTK1 had a significantly higher expression in poorly differentiated specimens than in well-differentiated specimens, which was consistent with Ki-67. PFTK1 was expressed in cell membrane, while cytoplasm, Ki-67 mainly in the nucleus. The result was shown in Fig 2. Then we investigated the correlation between PFTK1 and Ki-67 by Pearson’s correlation coefficient. We can see in Fig 3A that PFTK1 was positively associated with Ki-67 (Pearson’s γ = 0.833, *P*<0.001).

### PFTK1 is associated with adverse clinical characteristics and poor gastric tumor patients’ survival

Clinical association study was explored to analyze the correlation of PFTK1 with gastric cancer clinicopathological features among 161 tissues. The clinicopathological data were listed in Table 1. High expression of PFTK1 was related with tumor grade (*P* = 0.009), infiltration depth (*P* = 0.002), lymph node invasion (*P* = 0.000) as well as Ki-67 (*P* = 0.000). But there was no correlation between PFTK1 expression and other clinical variables, such as age, gender, nerve invasion, and TNM stage.

In addition, Kaplan-Meier analysis was used to analyze the association between PFTK1 expression and 161 patients’ survival. According to the survival curves, high expression of PFTK1 was obviously correlated with poor overall survival, while low expression of PFTK1 was correlated with better overall survival. And the median survival time and hazard ratio was showed in Fig 3B. Moreover, univariate analysis in gastric cancer tissues showed that tumor grade (*P* = 0.020), infiltration depth (*P* = 0.011), lymph node invasion (*P* = 0.011), TNM stage (P = 0.031), PFTK1 expression (*P* = 0.002), and Ki-67 expression (*P* = 0.013) were prognostic factors of overall survival (Table 2). Multivariate analysis using the Cox’s proportional hazards model suggested PFTK1 was an independent prognostic indicators of patients’ overall survival (*P* = 0.048, Table 3). To sum up, our results demonstrated that the expression of PFTK1 is significantly associated with clinical characteristics and poor overall survival.

**Table 2:** Contribution of various potential prognostic factors to survival by univariate analysis in 161 gastric specimens.

Characteristics	Total	Survival Status		χ^2^ value	P value
		Died	Alive		
Age				1.444	0.229
≤ 60	68	25	43		
60	93	43	50		
Gender				0.301	0.583
Female	53	24	29		
Male	108	44	64		
Tumor grade				7.832	0.020*
Well	16	3	13		
Moderate	82	31	51		
Poor and others	63	34	29		
Infiltration depth				8.945	0.011*
Inferiormucousemb-ranelayer	18	4	14		
Muscular layer	67	23	44		
Serous layer	76	41	35		
TNM stage				4.663	0.031*
I-II	58	18	40		
III-IV	103	50	53		
Lymph node				6.439	0.011*
Negative	16	2	14		
Positive	155	66	79		
Nerve invasion				0.879	0.348
Negative	96	37	59		
Positive	63	29	34		
PFTK1 expression				9.451	0.002*
Low	65	18	47		
High	96	50	46		
Ki-67 expression				6.188	0.013*
Low	63	19	44		
High	98	49	49		

Statistical analyses were performed by the Pearson χ^2^ test

* P 0.05 was considered significant

**Table 3:** Contribution of various potential prognostic factors to survival by Cox regression analysis in 161 gastric specimens.

	Hazard ratio	95% confidence interval	P value
Tumor grade	1.328	0.784–2.249	0.291
TNM stage	1.229	0.622–2.427	0.553
Lymph node	5.251	1.172–23.515	0.030*
PFTK1 expression	1.914	1.006–3.639	0.048*
Ki-67 expression	2.499	1.344–4.645	0.004*

Statistical analyses were performed by the Cox regression analysis

* P < 0.05 was considered significant

### The expression of PFTK1 in nontransfected and transfected gastric cancer cells

**Fig 4:**
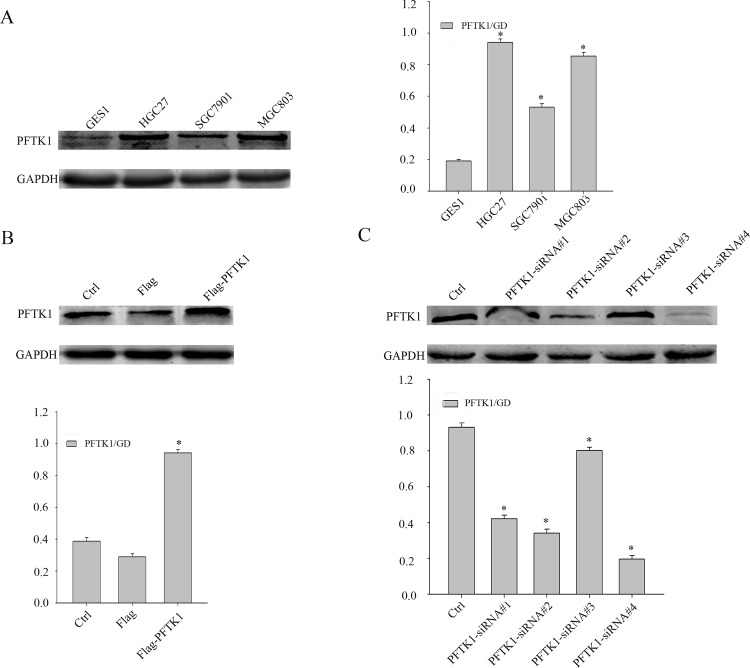
The expression of PFTK1 in nontransfected and transfected gastric cancer cells by western blot. (A) The PFTK1 protein level in SGC7901, MGC803, HGC27, GES1 gastric cell lines. (B) SGC7901 cells were transiently transfected with Flag-PFTK1 plasmid as described above for 48 h. Western blot analysis ectopic expression of PFTK1. (C) MGC803 cells were transiently transfected with PFTK1-siRNA#1, 2, 3, 4 for 48 h. Western blot analysis knockdown expression of PFTK1. The bar chart showed the ratio of PFTK1 protein to GADPH. Mean±SD of three independent experiments. (*P< 0.05).

As PFTK1 is overexpressed in gastric tumor tissues, then we analyzed expression of PFTK1 in cell lines including MGC803, SGC7901, HGC27, GES1. From Fig 4A, the expression of PFTK1 in normal gastric cell line GES1 was lower than in gastric cell lines MGC803, SGC7901, HGC27. For further study the function of PFTK1 in gastric cancer cells *in vitro,* MGC803 cells were transfected with PFTK1-siRNA and SGC7901 cells were transfected with Flag-PFTK1. Western blot was used to measure the PFTK1 expression. When Flag-PFTK1 was transfected in SGC7901, PFTK1 had a higher expression (Fig 4B). While PFTK1-siRNA was transfected in MGC803, PFTK1-siRNA#4 achieved the highest knockdown efficiency in comparison with other PFTK1-siRNA (Fig 4C). So we selected the Flag-PFTK1 and PFTK1-siRNA#4 to continue the following experiments.

### PFTK1 can promote cells migrate and invasive *in vitro*

**Fig 5:**
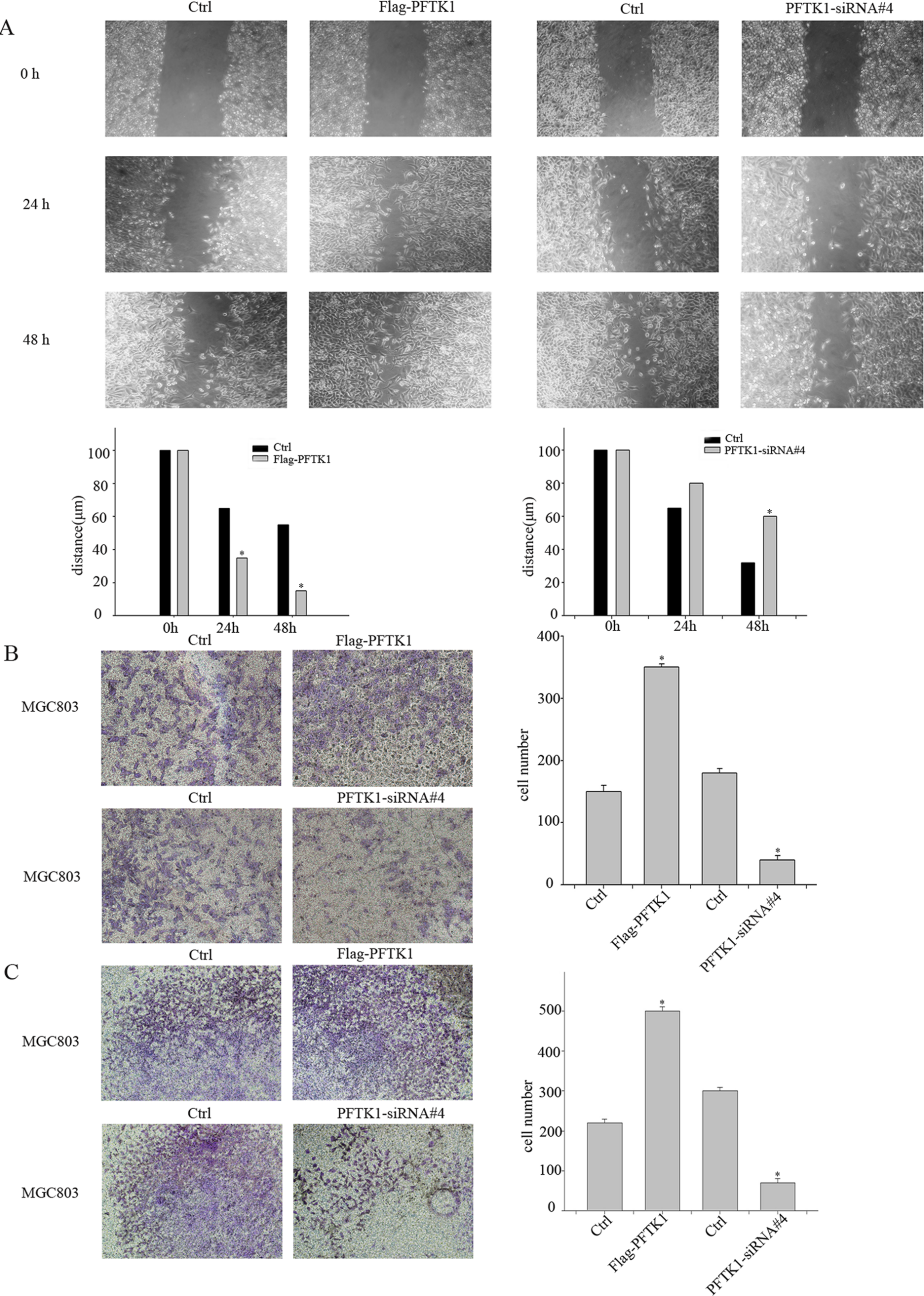
PFTK1 regulates the migration and invasion ability of gastric cancer cells. (A) Migration of cells transfected with Ctrl, Flag-PFTK1, PFTK1-siRNA#4was examined by wound-healing assays. The wound of cells was visualized at 0 h, 24 h, and 48 h. (B) Cell migration transfected with Ctrl, Flag-PFTK1, PFTK1-siRNA#4 was examined by trans-well assays. (C) The cell invasive ability transfected with Ctrl, Flag-PFTK1, PFTK1-siRNA#4 was examined with Matrigel cell culture chambers. The left part showed MGC803 cells transfected with Ctrl, Flag-PFTK1. The right part showed MGC803 cells transfected with Ctrl, PFTK1-siRNA#4. Mean±SD of three independent experiments (*P<0.05). These results show overexpression of PFTK1 can enhance cells migration and invasion, while knockdown PFTK1 reduce cells migration and invasion.

It is reported PFTK1 overexpression can increase CaD phosphorylation and enhance CaD to bind to F-actins, which promote hepatocellular carcinoma cells migration[16]. Given that PFTK1 was related to lymph node invasion in gastric cancer specimens, we studied whether PFTK1 could affect gastric cancer cells migration and invasion. Wound-healing assay and transwell assays showed that the migration of Flag-PFTK1 cells was notably increased in comparison with control cells. On the contrary, the migration of PFTK1-siRNA#4 cells were reduced in comparison with control cells (Fig 5A and 5B). Further, matrigel invasion assays also demonstrated that upregulation of PFTK1 by transfect Flag-PFTK1 could advance the invasive ability, and knockdown of PFTK1 could attenuate the invasive ability of gastric cancer cells (Fig 5C). Overall, we could come to a conclusion that PFTK1 promote migration and invasion of gastric cancer cells.

### Expression of PFTK1 promote proliferation of gastric cancer cells

**Fig 6:**
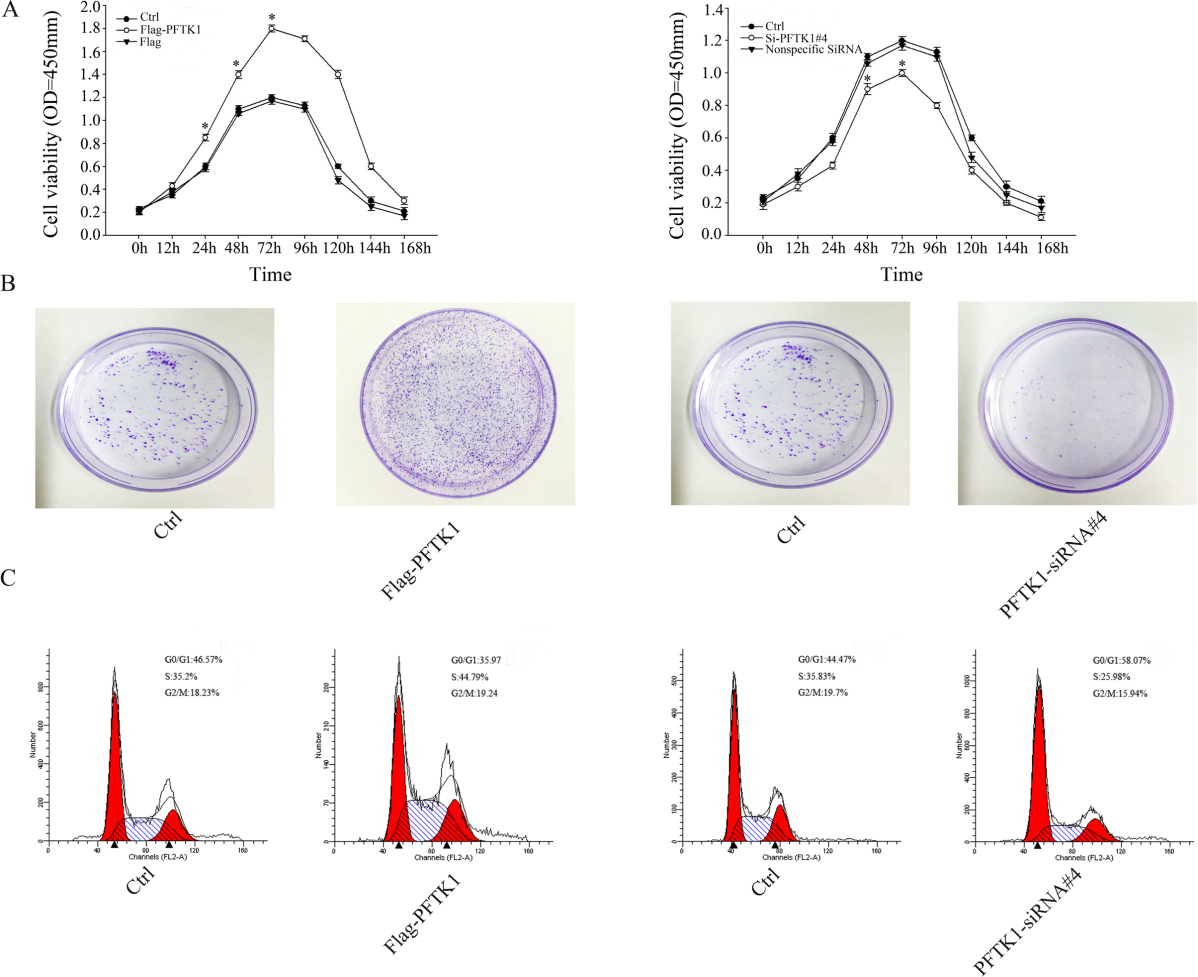
PFTK1 modulates the proliferation ability of gastric cancer cells. (A) *In vitro* Cell Counting Kit (CCK)-8 assay was used to examin cells growth by absorbance at 450 nm at the indicated time. (The data are means± SD from three independent experiments. *P < 0.05) (B) Colony formation analysis of MGC803 cells transfected with Ctrl, Flag-PFTK1, PFTK1-siRNA#4. (C) Cell cycle analysis was shown after transfected with Ctrl, Flag-PFTK1, PFTK1-siRNA#4 by flow cytometry. The Flag-PFTK1 cell number was decreased in G0/G1 phase and the corresponding increasing was observed in S phase compare with Ctrl. The PFTK1-siRNA#4 cell number was increased in G0/G1 phase and the corresponding reduction was observed in S phase compare with Ctrl. The left part showed MGC803 cells transfected with Ctrl, Flag-PFTK1. The right part showed MGC803 cells transfected with Ctrl, PFTK1-siRNA#4. These results show overexpression of PFTK1 can increase cells proliferation, while knockdown PFTK1 reduce cells proliferation.

According to the results presented above, PFTK1 was positively correlated with PCNA, Ki-67 expression and tumor grade, which were cells proliferation marker. We analyze the role of PFTK1 on regulating the proliferation ability. First, MGC803 cells were transfected with PFTK1-siRNA#4, Flag-PFTK1 and were determined CCK-8 assay. Overexpression of PFTK1 demonstrated to enhance gastric cells growth rate than control, while knockdown of PFTK1 showed the opposite result (Fig 6A). Consistent with enhancing cell growth rate following PFTK1 overexpression, colony-forming assay also showed it could increase colony formation. And knockdown of PFTK1 showed the same result with CCK-8 assay (Fig 6B). Finally, we explored the proliferation mechanism of PFTK1 by flow cytometric analysis,and data suggested that more gastric cancer cells were found in the S phase in the presence of ectopic PFTK1, less gastric cancer cells were found in the S phase in transfecting with PFTK1-siRNA#4 (Fig 6C). To summarize, these data demonstrated that PFTK1 participated in cell cycle regulation by accelerating G0/G1 –S transition.

### The signal pathway of PFTK1 in promoting proliferation and migration

**Fig 7:**
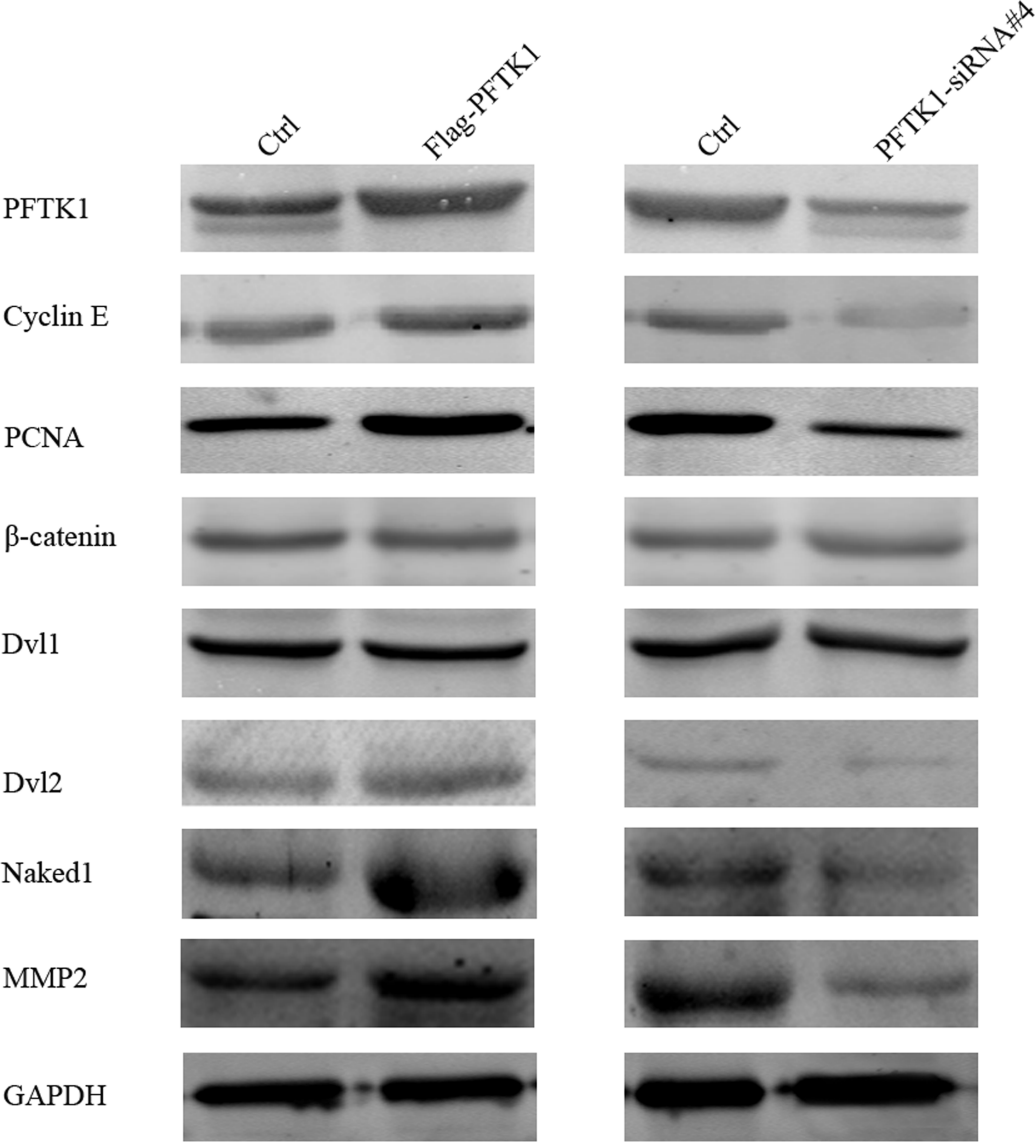
The effects of PFTK1 on the related signal pathway. The effects of PFTK1 on the expression of Cyclin E, PCNA, MMP2, Wnt related pathways such as β-catenin, Dvl1, Dvl2, Naked1 by western blot. Increasing PFTK1 expression promted the expression of Cyclin E, PCNA, Dvl2, Naked1, MMP2, but had not changed the expression of β-catenin, Dvl1. While knocking down endogenous PFTK1 expression showed the opposite result.

To further study the mechanism of which PFTK1 regulated gastric cancer cells proliferation, migration, invasion, we decided to detect the related protein changed by Western blot when PFTK1 was overexpressed or knockdown in MGC803. As reported, PFTK1 could activate noncanonical Wnt signaling in hepatocellular carcinoma [17]. Wnt signaling was related with tumors proliferation and migration. Upregulation of PFTK1 partly activated Dvl2, Naked1, MMP2 and elevated the expression of cell cycle regulator Cyclin E, but not changed Dvl1, β-catenin. What’s more, we found that knockdown of endogenous PFTK1 expression could decrease Dvl2, Naked1, MMP2, Cyclin E expression, but not changed Dvl1, β-catenin (Fig 7).

## Discussion

Being one of the highest incidence cancers in the world, the 5-year survival rate of gastric cancer is less than 20%–25% in the USA, Europe, and China because of easy to relapse and high metastasis rate in postoperative [2]. Helicobacter pylori infection, the change of oncogene and tumor suppressor genes, the regulation of cell cycle and telomerase activation are associated with gastric cancer [21]. Abnormal cell cycle regulation in the development of gastric cancer has been the topic of research in recent years. Accurate and strict regulation of cell cycle depends on some control factors including cell cycle dependent kinase (CDKs), cell cycle protein (Cyclins), and cell cycle dependent kinase inhibitors (CKIs). Everyone is familiar with 11 classic CDKs (CDK1-11), PFTK1 as a new cell cycle dependent kinase is found not along and the connection between PFTK1 and cancers is not much, also in gastric cancer [22].

PFTK1 was originally found in the nervous system of rats [23]. PFTK1 modulates oligodendrocyte differentiation via PI3K/AKT pathway [13]. The latest research reported that PFTK1 is upregulated in human esophageal cancer, hepatocellular carcinoma and associates with tumor growth, migration and invasion [14,24]. In our study, we explored whether the expression of PFTK1 was involved in gastric cancer cells proliferation, migration, and invasion. At first, we demonstrated that PFTK1 expressed higher in gastric cancer tissues than in adjacent nontumor tissues by western blot that in accordance with the results in other cancers (Fig 1). Subsequently, immunohistochemistry showed high expression of PFTK1 was related with tumor grade, infiltration depth, lymph node invasion as well as Ki-67 in 161 gastric cancer tissues. These findings prompted PFTK1 might affect gastric cancer proliferation and migration. Kaplan-Meier analysis revealed that PFTK1 predicted poor overall survival (Fig 3B). Multivariate analysis using the Cox’s proportional hazards model suggested PFTK1 was an independent prognostic indicators of patients’ overall survival. In order to further understand the regulatory effect of PFTK1 on gastric cells, MGC803 cells were transfected with PFTK1-siRNA#4, Flag-PFTK1 *in vitro* (Fig 4). Fig 6 showed PFTK1 could promote proliferation by CCK-8, colony-forming assay. At the same time, PFTK1 increased the G1—S phase transformation according to flow cytometric, coinciding with the finding of PFTK1 in hepatocellular carcinoma [10]. Cyclin E was one of the important regulatory factors during G1-S phase transformation and Cyclin E protein could define as a prognostic factor for patients with gastric cancer [25]. Interestingly, PFTK1 level was increased after transfecting with Flag-PFTK1 consistented with PCNA, MMP2 and Cyclin E (Fig 7).

In addition, we also examined whether PFTK1 could affect cells migration and invasion ability by wound-healing assay and transwell assays. The results showed that the overexpression of PFTK1 could enhance cells migration and invasion, which might be due to a change of downstream of the PFTK1 signal pathways (Fig 5). As reported in hepatocellular cancer, PFTK1 and/or CCNY alone could activate non-canonical Wnt signaling causing cells migration and invasion [17]. Wnt pathways included the canonical Wnt/β-catenin signaling and non-canonical Wnt signaling way(Wnt/Ca^2+^ and Wnt/Pcp). Wnt signaling way played an important role in the development of cancers and regulated cells proliferation, migration and invasion [26]. Subsequently, we found expression of proteins β-catenin, Dvl1, Dvl2, Naked1. Dvl2 and Naked1 were positively correlated with PFTK1 and the canonical Wnt/β-catenin related proteins β-catenin, Dvl1 had not changed (Fig 7). From the above, we therefore speculated that PFTK1 might promote migration and invasion through regulating non-canonical Wnt signaling. And there were also articles prompt non-canonical Wnt signaling promotes migration and invasion ability of gastric cancer cells [27,28].

In a word, the expression of PFTK1 significantly was increased in the human gastric cancer tissues. Overexpression or knockdown of PFTK1 by transfecting with Flag-PFTK1 or PFTK1-siRNA#4 obviously affected gastric cancer cells proliferation, migration and invasion. Our results at first time provide new evidence of PFTK1 in the development of gastric cancer and supply laboratory basis for clinical treatment. But further studies are needed to understand the precise mechanism of PFTK1 affecting proliferation, migration and invasion ability of gastric cancer cells.
